# Re-emergence of thiamine deficiency disease in the Pacific islands (2014–15): A case-control study

**DOI:** 10.1371/journal.pone.0198590

**Published:** 2018-06-07

**Authors:** Eric J. Nilles, Atarota Manaia, Bineta Ruaia, Clare Huppatz, Catherine Ward, Peter George, Christiaan Sies, Alessio Cangiano, James Sejvar, André Reiffer, Teatoa Tira

**Affiliations:** 1 Emerging Disease Surveillance and Response, Division of Pacific Technical Support, World Health Organization, Suva, Fiji; 2 Ministry of Health and Medical Services, Kuria, Kiribati; 3 Ministry of Health and Medical Services, Nawerewere, Tarawa, Kiribati; 4 Canterbury Health Laboratories, Christchurch, New Zealand; 5 University of Otago, Christchurch, New Zealand; 6 University of the South Pacific, Suva, Fiji; 7 National Center for Emerging and Zoonotic Infectious Diseases, Centers for Disease Control and Prevention, Atlanta, Georgia, United States of America; 8 World Health Organization, Nawerewere, Tarawa, Kiribati; Universidade de Sao Paulo, BRAZIL

## Abstract

**Background:**

From late 2014 multiple atolls in Kiribati reported an unusual and sometimes fatal illness. We conducted an investigation to identify the etiology of the outbreak on the most severely affected atoll, Kuria, and identified thiamine deficiency disease as the cause. Thiamine deficiency disease has not been reported in the Pacific islands for >5 decades. We present the epidemiological, clinical, and laboratory findings of the investigation.

**Methodology/Principal findings:**

We initially conducted detailed interviews and examinations on previously identified cases to characterize the unknown illness and develop a case definition. Active and passive surveillance was then conducted to identify additional cases. A questionnaire to identify potential risk factors and blood samples to assay biochemical indices were collected from cases and asymptomatic controls. Thiamine hydrochloride treatment was implemented and the response to treatment was systematically monitored using a five-point visual analogue scale and by assessing resolution of previously abnormal neurological examination findings. Risk factors and biochemical results were assessed by univariate and multivariate analyses. 69 cases were identified on Kuria (7% attack rate) including 34 confirmed and 35 unconfirmed. Most were adults (median age 28 years [range 0–62]) and 83% were male. Seven adult males and two infants died (13% case fatality rate). Resolution of objective clinical signs (78%) or symptoms (94%) were identified within one week of starting treatment. Risk factors included having a friend with thiamine deficiency disease and drinking kava; drinking yeast alcohol reduced the risk of disease. Higher chromium (p<0·001) but not thiamine deficiency (p = 0·66) or other biochemical indices were associated with disease by univariate analyses. Chromium (p<0·001) and thiamine deficiency (p = 0·02) were associated with disease by multivariate analysis.

**Conclusions/Significance:**

An outbreak of thiamine deficiency disease (beriberi) in Kiribati signals the re-emergence of a classic nutritional disease in the Pacific islands after five decades. Although treatment is safe and effective, the underlying reason for the re-emergence remains unknown. Chromium was highly and positively correlated with disease in this study raising questions about the potential role of factors other than thiamine in the biochemistry and pathophysiology of clinical disease.

## Background

Beriberi, or thiamine (vitamin B1) deficiency disease (TDD), became a serious public health problem, particularly in southeast Asia, in the late 19^th^ century after implementation of industrial rice milling that removes the thiamine-rich husk. By the early 20^th^ century, the link between polished rice and beriberi had been identified and public health interventions including fortification of rice largely eliminated beriberi as a common public health threat [1]. Outbreaks of beriberi are now rare and are usually reported in confined populations including prisoners or refugees, likely due to restricted diets with limited thiamine-rich food [2–7]. Less commonly, outbreaks occur in free-living populations, often suddenly and usually without well-characterized precipitating factors, raising questions as to the underlying drivers of disease [6–16]. Factors other than biochemical thiamine deficiency likely influence the epidemiology and clinical features of disease but these have not been well characterized [10,12,17,18].

TDD can be difficult to identify without a high index of suspicion because clinical features are varied, frequently subtle, and overlap with many other diseases. Well defined clinical types include dry beriberi, a peripheral neuropathy characterized by extremity paresthesias, weakness, muscle cramps, and areflexia; wet beriberi characterized by cardiac dysfunction presenting with edema, palpitations, and congestive heart failure; and Wernike-Korsakoff syndrome characterized by psychosis and encephalopathy, typically observed in developed countries in persons with severe alcohol dependence. Acute infantile beriberi affects infants aged 1–6 months that are breast-fed by thiamine deficient mothers; clinical features include dyspnea, cyanosis and other signs of heart failure. Young and middle-age adult males and pregnant and lactating women have the highest thiamine requirements and are most susceptible to deficiency. Response to treatment is usually rapid although neurologic features resolve more slowly and occasionally persist [5, 19]. Thiamine assays are rarely available in developing countries where disease is more common and diagnostic confirmation is usually based on response to treatment with thiamine [4–7, 11–16]. When thiamine diphosphate using high performance liquid chromatography and erythrocyte transketolase assays (the standard assays to assess biochemical thiamine deficiency) are performed, the correlation with clinical disease is usually poor [8,9,11,12,20–22].

From late 2014, clinicians in the Gilbert Islands in Kiribati identified an unknown and sometimes fatal neurological illness, primarily among previously healthy young and middle-age males. During January and February 2015 the Kiribati Ministry of Health and Medical Services (MHMS) and the World Health Organization (WHO) investigated an outbreak on Kuria, the most affected atoll. We describe the investigation which aimed to identify the cause of the outbreak, including the epidemiological, clinical and biochemical characteristics of cases, and the findings of a case-control study.

## Methods

This manuscript adheres to the STROBE checklist, a standard list of items recommended for inclusion in observational studies. Study data is available through Harvard Dataverse at https://doi.org/10.7910/DVN/YNIGU3.

### Setting

The Gilbert Island chain in the Republic of Kiribati (population 103,058) is composed of multiple scattered atolls and islands including Tarawa atoll, the country’s political, commercial and administrative centre with a population of 56,264 inhabitants, and Kuria atoll (Kuria), the site of the investigation, with a population of 980 residents and 190 households [23]. Residents on Kuria live a largely subsistence lifestyle with a diet based on reef and ocean fish, the red land crab (*Cardisoma carnifex)*, bread fruit, coconuts, pandanus, papaya, and swamp taro. Imported white rice is the staple carbohydrate. Kava and yeast alcohol are commonly consumed for their intoxicant effect. Kava is a mild sedative made of water and ground *Piper methysticum* root, which is imported from Fiji, Vanuatu or the Solomon Islands. Yeast alcohol is locally fermented water, sugar and yeast. Commercial alcohol is rarely available or consumed on Kuria.

### Identification and classification of cases

Between 15 and 17 January 2015 we conducted detailed medical interviews and examinations on eight affected and previously identified residents to characterize the illness and to define a case definition. We then identified cases with symptom onset between 1 January 2012 and 13 February 2015 by (i) interviewing medical providers and reviewing medical records, (ii) implementing prospective surveillance at both health facilities on Kuria and (iii) conducting active case finding in the community. A case with unconfirmed disease was anyone with paresthesias, pain, oedema, or weakness of the lower extremities without another reasonable explanation for the symptoms; or, an infant from one to six months of age who died with signs consistent with infantile beriberi (tachypnea, cyanosis, generalized oedema), without another reasonable explanation, and whose mother was a case with symptoms at the time of the infant’s deaths. A person with confirmed disease met the criteria for an unconfirmed case and also demonstrated rapid clinical improvement with treatment. Rapid clinical improvement was the resolution of a positive (abnormal) Heel Walk Test or Squat Test or complete or near-complete resolution of symptoms using a visual analogue scale within seven days of starting treatment.

### Case-control enrolment

A case-control study was conducted comparing 45 cases with beriberi to 105 controls without beriberi to identify risk factors and biochemical indices associated with disease. Cases and controls were enrolled by the joint MHMS/WHO outbreak investigation team between 15 January and 13 February 2015. Cases enrolled in the case-control study were confirmed or unconfirmed cases interviewed and examined by the investigation team. Two controls that were frequency matched by sex and age-group (15–30, 31–45, and 46–65 years) were enrolled per case based on a three-stage sampling framework. First, at the village level, the number of controls selected per village was proportional to the village population. Second, we selected every second household on the main road starting at the northernmost point of the village and moving South, or West to East. Third, at the individual level, we randomly selected one of six age-group sex categories (category) (male or female from one of the three age-groups) and selected an appropriate household member. If there was more than one household member in the category, we alternated between selecting the oldest and youngest. If there was no one in the correct category, we randomly selected another category, etc. If no adult resident was home or if there was not a resident in a required category, we selected the previous household and then returned to the original sampling frame. This process was continued until the pre-determined numbers of controls per category for each village were enrolled. One control was selected per household. Exclusion criteria included refusal to participate, a case living in the household or any symptom listed in the case definition at the time of household visit. A questionnaire that included questions on demographics, occupation, current symptoms, 27 dietary variables and other potential exposures (drinking well water, kava, yeast alcohol, tobacco use) was developed by the investigation team and administered to all cases and controls. The questionnaire was developed by the investigation team based on common local foods and other potential exposures and risk factors.

### Cross-sectional study

We collected blood samples from residents on Tarawa atoll, an urban atoll without any reported beriberi cases. Participants were frequency matched to cases by age-group and sex and identified from two villages (Bairiki and Bikenibeu) that were randomly selected from six villages with >3000 residents [23]. We identified households for enrolment by visiting every third household starting from the center of each village as defined by the investigation team, and moving from East to West; otherwise enrolment adhered to the methods described for control enrolment.

### Clinical and laboratory investigations

Physical examinations that included a focused neurological examination (gross motor; sensation to light touch; deep tendon reflexes; squat, heel- and toe-walk tests) were conducted on cases and a convenience sample of controls by a study author at the time of the investigation. Information on fatal cases was obtained from medical records and interviews with family members and health care providers. Blood samples were collected at the time of enrolment from cases, every second control, and participants enrolled on Tarawa, in blood collection tubes containing a clot activator and EDTA. Samples were immediately placed on ice, and EDTA samples were frozen within two hours. Blood collected in vacutainers containing clot activator was left to stand, clot, and separate for ~2 hours at which time sera was extracted into serum separator tubes and frozen. Urine from a convenience sample of cases was collected in urine specimen containers and frozen. Samples were shipped frozen to Canterbury Health Laboratories, Wellington, New Zealand (CHL). We performed biochemical assays on whole blood or serum for heavy metals (mercury, arsenic, lead) or micronutrients (calcium, chromium, copper, folate, magnesium, phosphate, thiamine, selenium, vitamins B6, B12, and E) that may be associated with weakness or neuropathy [24]. Whole blood thiamine diphosphate and chromium were assessed using high performance liquid chromatography (HPLC) with fluorescence detection and inductively coupled plasma mass spectrometry, respectively; the other assays performed and quality control measures for all assays are available at www.labnet.health.nz/testmanager. Reference intervals (normal range values) are those used by Canterbury Health Laboratories. Biochemical thiamine deficiency was defined as a thiamine diphosphate level below the normal range (180 mmol/L) regardless of presence or absence of clinical symptoms [24]. The case status of samples was not known by laboratory scientists performing the assays.

### Response to treatment

After demonstrating a rapid response to thiamine in the initial case treated, all suspected cases with persistent symptoms were offered 100 mg thiamine hydrochloride by mouth daily or for cases of severe disease 100 mg intramuscular daily for 1–3 days followed by 100 mg oral daily for between 3–6 weeks. The optimal duration of treatment has not been well defined and ranges from three days to six-weeks have been reported [2, 5, 20]. Our treatment protocol required a minimum of three weeks of thiamine supplementation, but in the event of persistent symptoms treatment was extended until complete symptom resolution or for six-weeks of treatment whichever was first. Response to treatment was monitored objectively by repeating specific elements of the neurological examination that are frequently abnormal in beriberi (Squat Test, Heel-Walk Test, lower extremity hypo- or areflexia) and subjectively by self-reported symptom resolution using a 5-point visual analogue scale, a tool validated to assess subjective symptoms [25]. The visual analogue scale and examination were repeated every 1–3 days for the first seven days of treatment. For cases reporting persistent symptoms after seven days, the visual analogue scale was repeated weekly for an additional six weeks or until resolution of symptoms. A positive Squat Test was defined as an inability to rise from a squatting to a standing position without assistance. A positive Heel-Walk Test was an inability to walk 10 meters while maintaining full ankle dorsiflexion and walking only on the heels.

### Statistical analyses

Population figures from the 2010 census were used to calculate rates of disease [23]. Risk factors and laboratory covariates were initially assessed using χ^2^ to compare proportions and ANOVA or Kruskal-Wallis tests to compare means. Risk factors with a p-value <0·10, sex, and age-group were included in a logistic regression model and independent risk factors for disease were assessed by backward elimination, and excluded if the p-value was >0.05 and did not meaningfully alter the point estimates of the remaining variables. Laboratory covariates correlated with disease with a p-value <0·10 by either proportion or mean, and biochemical thiamine deficiency, were included in a logistic regression model using the same methods. All analyses were performed with IBM SPSS version 22·0.

### Ethical approval

No human subjects review committee exists in Kiribati and the MHMS authorized this study. The WHO Regional Office for the Western Pacific Ethics Research Committee determined this investigation to be exempt from review given the investigation and response was related to an acute public health event requiring immediate interventions. Prior to enrolment, witnessed verbal informed consent was obtained and documented for all study subjects or their legal guardian if < 18 years, a process approved by the Kiribati MOHM and the WHO Ethics Research Committee. Verbal versus written consent was obtained due to low literacy rates.

## Results

### Case finding

**Fig 1:**
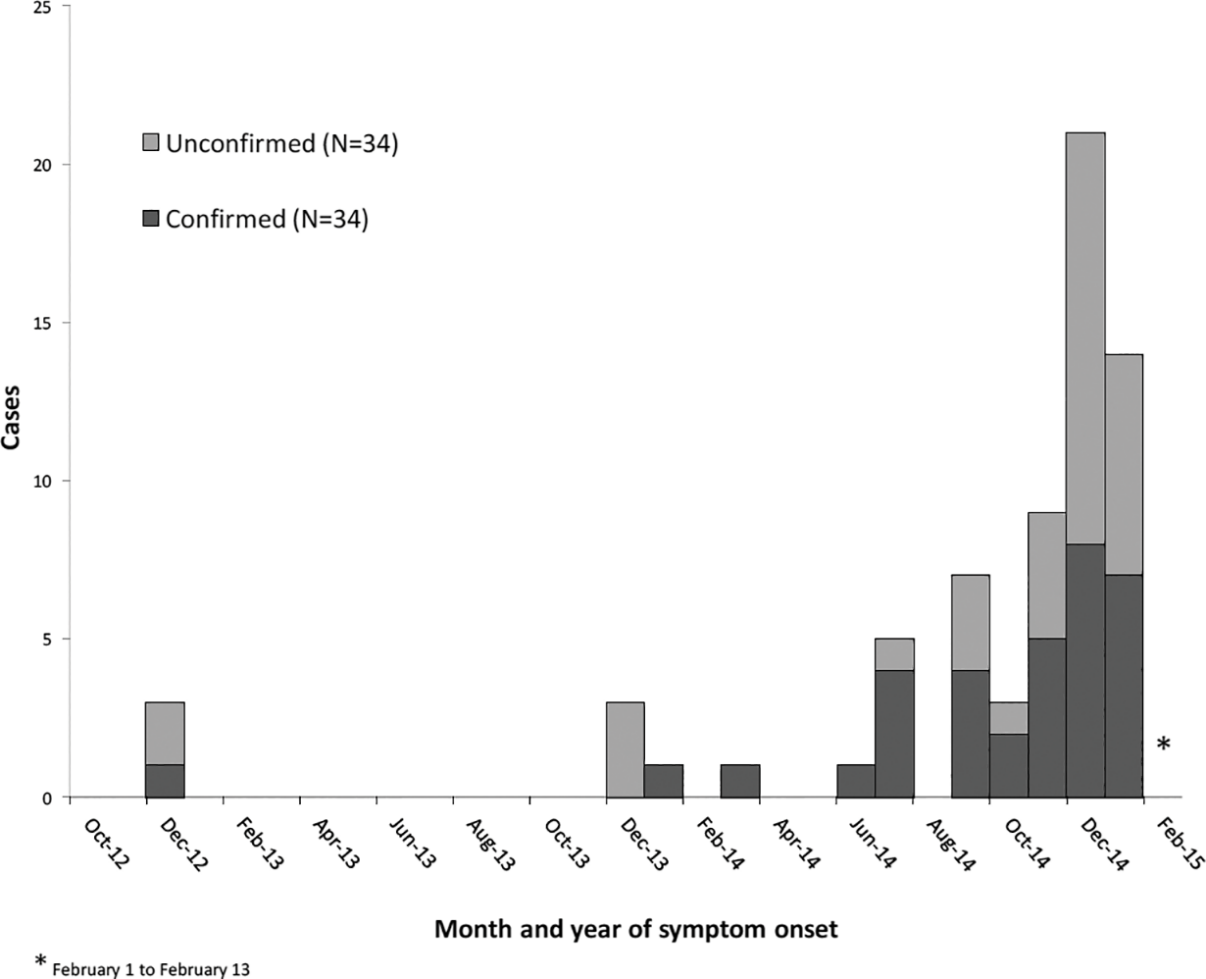
Monthly cases of confirmed and unconfirmed thiamine deficiency disease by month and year of symptom onset on Kuria, Kiribati, during the period from 1 October 2012 to 13 February 2015.

A total of 69 cases including 34 confirmed and 35 unconfirmed cases were identified on Kuria (attack rate 7%) and are included in the descriptive analysis. The median age was 28 years (range 0–62) and 83% were male. The initial cases developed symptom during December 2012. The outbreak peaked in December 2014, and declined during January 2015 (Fig 1). The attack rate for males was 10·8% (57/508) with the highest attack rate observed in males from 15–49 years (19·8%, 49/247). Nine cases died (case fatality rate 13%) including seven adult males with a median age of 23 years (range 22–49 years) and two infants aged eight and eleven weeks. Of ten adult females with complete information, six were lactating and two were pregnant at the time of symptom-onset. The mothers of both fatal infant cases were confirmed beriberi cases that developed symptoms within two weeks preceding the infants’ deaths. Clinical features are presented in Table 1.

**Table 1:** Clinical features of cases and controls, Kuria, 2015.

Clinical Feature	Cases (n = 52)*		Controls (n = 18)*	
	Obs	%	Obs	%
**Distribution**				
Upper and lower extremity	23	44%	0	0%
Lower extremity only	29	56%	0	0%
Bilateral	50	96%	0	0%
**Extremity symptoms [Included in case definition]**	** **	** **		
Extremity weakness	39	75%	0	0%
Extremity paresthesias	39	75%	0	0%
Extremity numbness	39	75%	0	0%
Extremity pain	28	54%	0	0%
Extremity oedema (history)	16	31%	0	0%
**Other symptoms [Not included in case definition]**	** **	** **		
Palpitations or racing heart	24	46%	2	11%
Shortness of breath	24	46%	2	11%
Epigastric pain	21	40%	1	6%
Constipation	16	31%	4	22%
Anorexia	11	21%	0	0%
Nausea	10	19%	1	6%
Chest pain	5	10%	0	0%
Vomiting	5	10%	0	0%
Diarrhea	1	2%	0	0%
**Clinical signs—General**	** **	** **		
Temperature ≥ 38.0° C^¥^	0	0%		
Tachypnea (respiratory rate ≥ 20/min)^#^	17	49%	11	69%
Tachycardia (pulse > 100/min) ^#^	5	14%	2	13%
Systolic flow murmur on cardiac examination ^†^	10	20%	1	6%
Abnormal pulmonary examination^¶^*^†^	2	4%	0	0%
Edema, lower extremity	13	25%	0	0%
**Neurological tests—General**	** **	** **		
Positive Heel-Walk Test ^Ʊ^	30	58%	0	0%
Positive Squat Test ‡	21	54%		
Abnormal toe-walking test^†^	8	16%	0	0%
Gross motor weakness lower extremities (<5/5)^†^§	8	16%	0	0%
**Neurological tests—Deep tendon reflexes**				
Patellar—areflexia^Ʊ^	21	40%	0	0%
Patellar—hyporeflexia ^Ʊ^	11	21%	1	6%
Patellar—hyperreflexia^Ʊ^	2	4%	0	0%
Achilles—areflexia^Ʊ^	22	42%	0	0%
Achilles—hyporeflexia ^Ʊ^	10	19%	1	6%
Achilles—hyperreflexia^Ʊ^	1	2%	0	0%
Biceps—areflexia ^Ʊ^	10	19%	0	0%
Biceps—hyporeflexia ^Ʊ^	8	15%	0	0%
Biceps—hyperreflexia^Ʊ^	0	0%	0	0%

Obs = Observations

* Cases with complete or near-complete examination data (includes 34 confirmed and 18 unconfirmed cases); controls were a convenience sample

¥ Temperature not measured in controls

¶ Decreased breath sounds over the left lower lobe (both cases); no rales or rhonchi.

# 17 cases and 2 controls missing data

† 1 case and 1 control missing data

‡ 13 cases missing data, not assessed for controls

Ʊ 1 control missing data

§ Includes 7 cases with 4/5 and one case with 3/5 motor strength of multiple lower extremity motor groups

### Response to treatment

Data presented is for 36 cases treated and monitored on Kuria by the investigation team. Of the remaining 33 cases, nine died, 11 were lost to follow-up, 12 had spontaneous resolution of symptoms, and one was treated with thiamine on Tarawa with rapid resolution of symptoms reported. The mean and median duration of symptoms at the time of investigation and prior to treatment was 96 days (Standard Deviation [SD]: 88) and 63 days (range 7–378). Within seven days of starting treatment, 34 (94%) of 36 cases reported complete or near-complete resolution of symptoms: 19 (53%) reported complete resolution of symptoms; 15 (42%) reported 80% resolution, one (3%) reported 60% resolution, and one (3%) reported 40% resolution. After treatment, 11 (65%) of 17 cases that previously failed the Squat Test could complete the test within three days and 13 (77%) within seven days. Among 22 cases that were unable to complete the Heel Walk Test prior to treatment, 12 (55%) and 16 (73%) could successfully complete the exercise within three and seven-days of treatment respectively. Improvement of abnormal deep tendon reflexes after one week of treatment was observed in only one treated case. All cases reported complete resolution of symptoms by VAS within six weeks of starting treatment. None of the treated cases required or received additional pharmacological treatment, as defined by local health providers.

### Risk factors for disease

A total of 45 cases (34 confirmed and 11 unconfirmed) and 105 controls were enrolled in the case-control study. Controls were enrolled from approximately 55% of all households on Kuria. No selected individuals refused to participate as a control. Characteristics of cases and controls are listed in Table 2 and crude and adjusted odds ratios for disease in Table 3. Having a friend with beriberi (OR 8·7 [95% CI 2·9–25·7]) and drinking kava one or more times weekly (OR 3·2 [95% CI 1·3–7·9]) predicted disease; drinking yeast alcohol one or more times weekly reduced the risk of disease (OR 0·1 [95% CI 0·03–0·6]).

**Table 2:** Demographics and mean thiamine diphosphate and chromium values in Kuria cases and controls and Tarawa participants.

Variable	Kuria				Tarawa	
	Cases (n = 45)		Controls (n = 105)		Participants (n = 64)	
Median age (range)	30	16–54	30	15–63	28	16–63
Female (%)	8	18	10	10	7	11
Mean TDP nmol/L (SD)†	183	59	190	63	244	62
Mean chromium nmol/L (SD)‡	10·9	2·9	7·6	2·6	6·9	0·9

† TDP (thiamine diphosphate) results for 36 cases (31 confirmed, 5 unconfirmed), 59 controls, 51 Tarawa participants (3 cases excluded due to blood collected after initiation of thiamine treatment; post-treatment levels were 152, 211, 213)

‡ Chromium results for 39 cases (33 confirmed, 6 unconfirmed), 59 controls, 51 Tarawa participants

**Table 3:** Crude and adjusted odds ratios and 95% CIs for risk factors for thiamine deficiency disease.

Risk factor*	Cases (n = 45)		Controls (n = 105)		Odds Ratio (95% CI)		Adjusted p-value
	Obs	%	Obs	%	Crude	Adjusted	
Collect coconuts for income	30	67%	89	85%	0·4 (0·2–0·8)	**	
Fisherman	26	58%	82	78%	0·4 (0·2–0·8)	**	
Store owner or worker	6	13%	3	3%	5·2 (1·2–21·9)	**	
Having friend with disease	19	42%	12	11%	5·9 (2·5–13·8)	8·7 (2·9–25·7)	<0·0001
**Consumption†**							
Raw fish	36	80%	68	65%	2·2 (0·9–5·0)	**	
Crab	17	38%	59	56%	0·5 (0·2–1·0)	**	
Coconut flesh	33	73%	91	87%	0·4 (0·2–1·0)	**	
Yaqona (kava)	27	60%	36	35%	2·9 (1·4–5·9)	3·2 (1·3–7·9)	0·01
Yeast alcohol	4	9%	31	30%	0·2 (0·1–0·7)	0·1 (0·03–0·6)	0·01
Sour toddy‡	4	9%	23	22%	0·3 (0·1–1·1)	**	
Chew tobacco	0	0%	11	10%	Undefined	**	

Obs = observations

*Includes occupation, exposure to persons with disease, and diet characteristics

**Non-significant in final regression model

†Consumption or use one or more times per week versus less than one time per week

‡ Fermented coconut palm sap

### Laboratory results

Cases versus control: The proportion of cases and controls with biochemical thiamine deficiency (p = 0·66) or other micronutrient deficiencies were similar when evaluated by univariate analyses (Table 4). Elevated mercury, arsenic and selenium levels were identified in most samples but no differences were identified between cases and controls. When evaluating the data by means rather than proportions, the results were similar except for chromium which, despite most samples being within the normal range, was consistently higher in cases compared to controls (mean 10·9 nmol/L [SD 2·9] versus 7·6 nmol/L [SD 2·6]) (p<0·0001). The final model of biochemical covariates included thiamine deficiency (OR 3·8 [95% CI 1·2–11·8], p = 0·02) and elevated chromium (OR 1·7 per mmol/L [95% CI 1·4–2·1], p<0·001).

**Fig 2:**
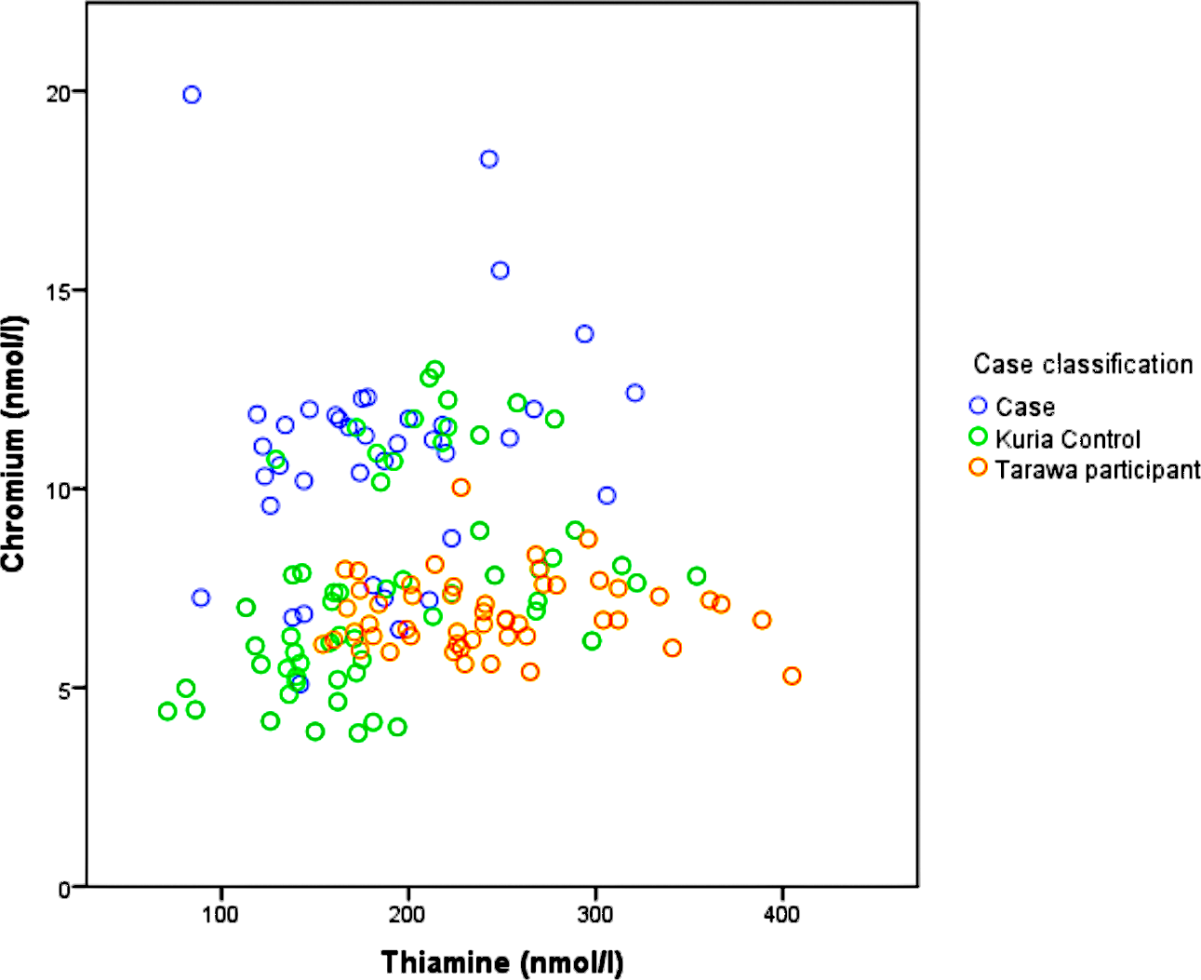
Thiamine diphosphate and chromium in cases, controls and Tarawa participants, 2015.

Kuria versus Tarawa: The prevalence of biochemical thiamine deficiency was higher on Kuria (50/95, 52%) than Tarawa (9/51, 18%) (p<0·001). When excluding cases and comparing only Kuria controls to Tarawa participants, the findings were similar (30/59 (51%) versus 9/51 (18%) (p<0·001)). The mean chromium level was higher on Kuria than Tarawa (8·9 nmol/L [SD 3·2] versus 6·9 nmol/L [SD 0·9]) (p<0·001); this was largely due to the higher chromium levels among cases and when evaluating only Kuria controls to Tarawa the difference was not significant (7·6 nmol/L [SD 2·6] versus 6·9 nmol/L [SD 0·9], p = 0·08) (Fig 2).

**Table 4:** Biochemical results for cases and controls on Kuria, 2015.

Assay	Metal/ micronutrient	Category (normal range)*	Cases Kuria		Controls Kuria		Odds ratio (95% CI) ◊	p-value
			No.	%	No.	%		
**Whole blood†**	Thiamine§	Normal (180–300 nmol/L)	16	44%	29	49%	Ref	0·66
		Deficient	20	56%	30	51%	1·2 (0·5–2·8)	
	Chromium	Normal (1–12 nmol/L)	35	90%	57	97%	Ref	0·17
		Elevated	4	10%	2	3%	3·3 (0·6–18·7)	
	Selenium	Normal (0·6–1·8 umol/L)	0	0%	0	0%	Ref	
		Elevated	39	100%	59	100%		
	Mercury	Normal (< 50 nmol/L)	5	13%	3	5%	Ref	0·18
		Elevated	34	87%	56	95%	0·4 (0·1–1·6)	
	Arsenic	Normal (0–0·16 μmol/L)	12	31%	20	34%	Ref	0·75
		Elevated	27	69%	39	66%	1·1 (0·5–2·7)	
	Lead	Normal (0–0·5 μmol/L)	39	100%	59	100%	Ref	
		Elevated	0	0%	0	0%		
	Vitamin B6	Deficient	3	8%	0	0%		0·76
		Normal (35–107 nmol/L)	35	90%	56	95%	Ref	
		Elevated	1	3%	3	5%		
**Urine‡**	Copper	Normal (< 1 μmol/L)	24	100%			Ref	
		Deficient	0	0%				
**Serum¥**	Vitamin B12	Normal (130–650 pmol/L)	35	100%	14	100%	Ref	
		Deficient	0	0%	0	0%		
	Vitamin E	Normal (17–32 μmol/L)	34	97%	13	93%	Ref	0·51
		Deficient	1	3%	1	7%	0·4 (0·0–6·6)	
	Calcium	Normal (2·2–2·6 mmol/L)	35	100%	14	100%	Ref	
		Deficient	0	0%	0	0%		
	Phosphate	Normal (0·8–1·5 mmol/L)	35	100%	14	100%	Ref	
		Deficient	0	0%	0	0%		
	Magnesium	Normal (0·6–1·2 mmol/L)	35	100%	14	100%	Ref	
		Deficient	0	0%	0	0%		
	Folate	Normal (> 8 nmol/L)	22	63%	11	69%	Ref	0·28
		Deficient	13	36%	3	31%	2·2 (0·5–9·2)	

* Biochemical assays and location of participants with levels exceeding the normal range but classified as normal in this table include thiamine (2 cases, 3 Kuria controls), Vitamin B12 (27 cases, 11 Kuria controls), Vitamin E (19 cases, 6 Kuria controls) phosphate (14 cases, 7 Kuria controls).

◊ Bivariate binomial logistic regressions performed with metals/micronutrients as dependent variables (0 = normal, 1 = elevated/deficient) and case classification as independent variable (Reference category = Kuria control)

† 39 cases and 59 control whole blood samples

‡ 24 cases and no control urine samples

¥ 35 cases and 14 control serum samples

§ 3 cases excluded that had blood collected after initiation of thiamine treatment (post-treatment levels were 152, 211, 213)

## Discussion

Since Patrick Manson described beriberi as a “tropical pathological puzzle” in the late 19^th^ century, many aspects of the disease have been elucidated leading to important public health interventions and widespread control. But endemic disease and outbreaks continue to be reported [26,27] and important knowledge gaps remain. For example, as with our study, compelling evidence is rarely reported to explain the timing, magnitude or spatial distribution of outbreaks in free living populations. Similarly, although beriberi and TDD are often used interchangeably, this and prior studies suggest that TDD correlates poorly with biochemical thiamine deficiency.

Our study identified yeast alcohol, kava, and having a friend with the disease as independently associated with beriberi. Yeast is thiamine-rich and regular consumption of yeast alcohol would be expected to decrease the risk of TDD. Being a friend of a case was a powerful predictor of disease and implies shared activities, practices, diets or other exposures requiring further investigation. Kava was also strongly associated with disease and additional dose response analyses indicated that consumption more than once per week doubled the risk of TDD and consumption more than four times per week increased the risk eight-fold, compared to consuming less than once per week. We think it improbable, however, that an intrinsic biochemical constituent of *Piper methysticum* root or routine behaviours associated with kava-drinking were important in this outbreak given Kava is frequently and widely consumed in the Pacific islands and no cases of beriberi have been documented in the Pacific since the 1960s. Conversely, introduction of a contaminant on Kuria–and perhaps more widely—during the preparation or consumption of kava that precipitates disease is plausible. Notably, cooked or raw fish, the primary sources of dietary protein on Kuria and most of Kiribati, were not associated with beriberi in this study, an important negative finding given that certain species of fish (and some plants) contain thiaminase compounds that inactivate thiamine by splitting the molecule into inactive components. Similarly, thiaminase activity was not detected in samples of commercial ground kava from Fiji and Vanuatu (Pers. Comm., C. Kraft, Cornell University). However, other plant or bacterial sources of thiaminase that were not measured in this study could potentially be responsible for the outbreak.

Consistent with prior controlled field studies [8,9,11,12,20,22] our study did not identify a direct correlation between biochemical thiamine deficiency and disease despite documenting a rapid clinical response to treatment with thiamine hydrochloride. Although several hypotheses have been proposed to explain the poor correlation [11,12], the most compelling appears to be that TDD is multifactorial, and additional factors precipitate or contribute to overt clinical disease [5,12,27].

Our study findings align with a multifactorial hypothesis, and suggest that chromium may be an important factor in disease manifestation. Chromium was strongly correlated with disease by crude and adjusted analyses (both p<0·001) and thiamine correlated with disease (p = 0·02) when adjusting for chromium, with improved model fit when thiamine was included. The biochemistry of chromium has not been well characterized and its role in human physiology continues to be debated [28], but some non-human laboratory studies suggest elevated chromium may impair or interrupt thiamine activity [28,29]. Chromium is a component in stainless steel and ubiquitous in the developed and developing world, often in cookware. Prior to the widespread use of stainless steel in the early and mid-20^th^ Century, chromium was commonly used in dyes and tanning salts. To our knowledge, no prior studies have implicated chromium in the pathophysiology of beriberi or TDD in humans.

There are multiple inherent limitations, however, when extrapolating cause and effect from associations, particularly in the setting of naturally occurring events outside a structured experimental setting; an observational association such as the correlation between TDD and chromium presented in this paper should be considered hypothesis-generating only. Despite this caveat, if our findings are replicated they may provide important insights into the biochemistry of thiamine deficiency disease, with equally important implications for disease prevention. Another study limitation includes an inability to verify that cases included in the study were beriberi (or TDD) and not another condition. However, we are confident that the majority of cases were in fact beriberi given (i) the classical epidemiological characteristics of beriberi (young males, pregnant and lactating females, and infants born to mothers with features of clinical beriberi), (ii) a population with widespread biochemical thiamine deficiency heavily dependent on rice as the core staple carbohydrate, (iii) classical clinical features of beriberi, and (iv) rapid and consistent response to treatment in >90% of treated cases.

Although our outbreak investigation was limited to Kuria, reports of an unusual illness consistent with TDD have been reported from four other atolls in the Gilbert Islands. An unpublished MHMS investigation from July 2015 reported 38 beriberi cases from Butaritari Island in the Northern Gilbert Islands, all of which responded rapidly to treatment; and 24 new cases that responded to treatment were reported from Kuria between February and November 2015. The underlying reason for the temporal-spatial nature of the outbreak remains unknown but these reports do indicate ongoing re-emergence of a classic nutritional disease with important public health implications. Increased awareness of and surveillance for thiamine deficiency disease should be considered in the Pacific islands and other regions at risk. If suspected cases are identified, treatment with thiamine is safe, effective, cheap and diagnostic, and should be implemented promptly. Treatment should not be delayed pending biochemical confirmation of thiamine deficiency.
